# Impact Factor for *WestJEM*

**DOI:** 10.5811/westjem.2017.10.36756

**Published:** 2017-10-16

**Authors:** Mark I. Langdorf

**Affiliations:** University of California, Irvine School of Medicine, Department of Emergency Medicine, Orange, California

I want to take this opportunity to update our readers, reviewers, and supporters regarding the growth and stature of the *Western Journal of Emergency Medicine: Integrating Emergency Care with Population Health* (*West*JEM). We continue to grow and thrive, extending our scope and reach throughout the nation and the world.

One of the measures of a successful journal is its ability to make its published research available to other researchers, policy makers and thought leaders through robust indexing. *West*JEM is indexed in all the world’s sources, including MEDLINE/Index Medicus, PubMed, PubMed Central, Europe PubMed Central, Embase, EBSCO/CINAHL, SCOPUS, HINARI (World Health Organization journal list) and Clarivate (formerly Thomson-Reuters) Emerging Sources Index. As an open access journal, we are members of the Directory of Open Access Journals, which indexes our abstracts.

The journal’s two-year impact factor from Scimago Journal and Country Rank (SJR) is 1.136 for 2016. This ranks us 21^st^ of 76 journal titles in emergency medicine. This is equivalent to the Clarivate (former Thomson-Reuters) impact factor that is commonly used to gauge a journal’s influence. This ranking can be found at: http://www.scimagojr.com/journalrank.php?category=2711&area=2700&order=cpd&ord=desc&page=1&total_size=76 and lists *West*JEM as the 3rd ranked (of 12), fully open-access journal in the specialty.

The trend for impact factor can be found at: http://www.scimagojr.com/journalsearch.php?q=19900193277&tip=sid&clean=0 (Image 1).

Our three-year impact factor from Scopus 2016 CiteScore journal metrics is 0.95 (Image 2). This ranks us 25^th^ of 75 journal titles in emergency medicine, and rising.

Comparison to other journals in the specialty can be found here: https://www.scopus.com/sourceid/19900193277?origin=sbrowse#tabs=1

And specific *West*JEM score can be found at: https://www.scopus.com/sourceid/19900193277?origin=sbrowse#tabs=0

Why does this matter? The more robustly indexed a journal, the more likely a researcher interested in your work is to find, and cite, your article. Given that *West*JEM is one of a few fully open-access emergency medicine journals in the world, the full published paper is available to anyone with an internet connection without charge. Furthermore, authors published in *West*JEM retain their own copyright, and are not asked to sign over rights to their intellectual property as a condition of publication, like subscription-based journals. *West*JEM’s article processing fee upon acceptance is the lowest among open-access journals. There is never a fee to submit a paper.

Each issue of *West*JEM is distributed electronically to more than 23,000 readers, researchers and clinicians, and mailed to more than 4,000. Content is also distributed through Medscape, with some 250,000 hits per year (http://www.medscape.com/viewpublication/21595). There were 1.8 million article views and downloads of *West*JEM papers in 2016. Clearly, distribution is wide and vast, offering the greatest chance of your work being cited.

Immediate impact on social media of *West*JEM papers is tracked through Altmetrics, with our highest impact papers achieving more than 400 notices by news outlets, tweets, Facebook reposts, and academic reader inclusions. https://www.altmetric.com/details/9119550

Publishing in *West*JEM is mainstream. The scientific quality of papers continues to increase, as the journal contributes to the greater good of the specialty. With the population health and public policy niche of the journal, this ultimately benefits our community and society.

Thank you for your faith in our efforts.

## Figures and Tables

**Figure 1:**
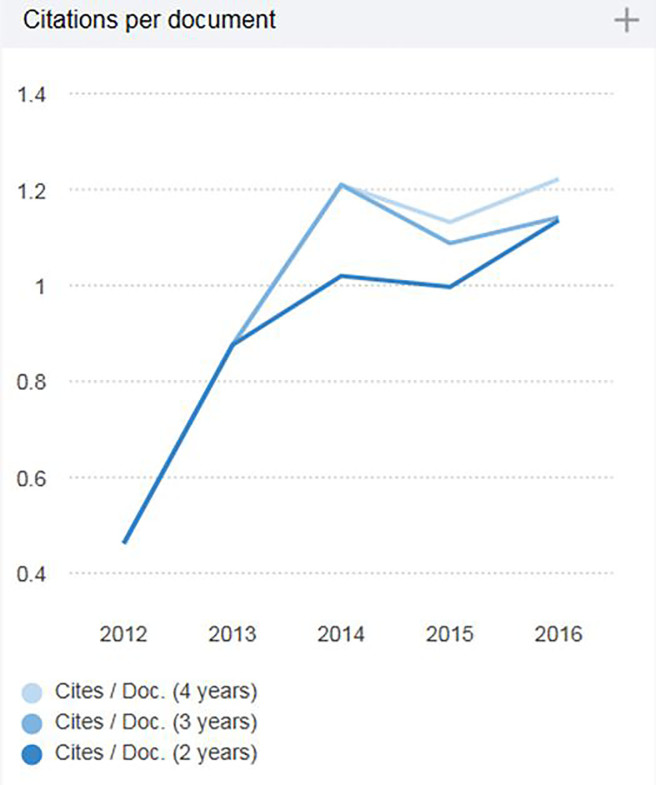
Trend of two-, three-, and four-year impact factor for *Western Journal of Emergency Medicine*. These are citations in index year of journal documents in previous two, three and four calendar years. Two-year impact factor is the same as Clarivate (formerly Thomson-Reuters) two-year impact factor.

**Figure 2:**
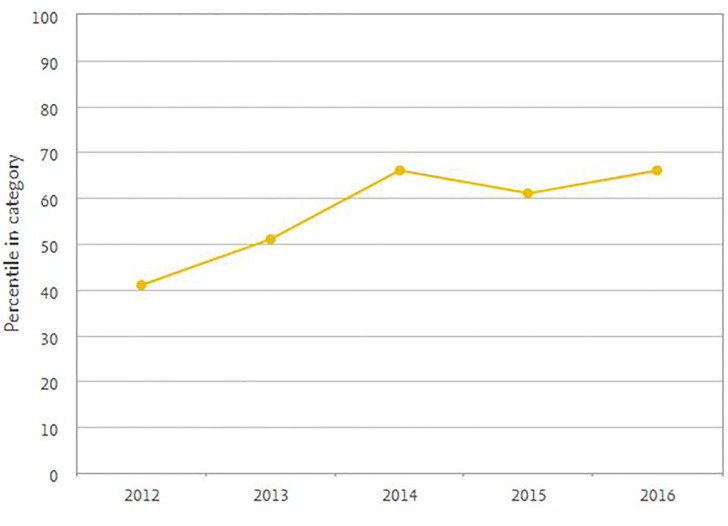
Percentile trend compared with 75 journals in emergency medicine in Scopus CiteScore for *Western Journal of Emergency Medicine* (higher is better).

